# *Dermestes* (*s.str.*) *haemorrhoidalis* (Coleoptera: Dermestidae)—The Most Frequent Species on Mummified Human Corpses in Indoor Conditions? Three Cases from Southwestern Poland

**DOI:** 10.3390/insects14010023

**Published:** 2022-12-25

**Authors:** Marcin Kadej, Łukasz Szleszkowski, Agata Thannhäuser, Tomasz Jurek

**Affiliations:** 1 Centre for Forensic Biology and Entomology, Department of Biology, Evolution and Conservation of Invertebrates, University of Wrocław, Ul. Przybyszewskiego 65, PL-51-148 Wrocław, Poland; 2 Department of Forensic Medicine, Wroclaw Medical University, Ul. Mikulicza-Radeckiego 4, PL-50-345 Wrocław, Poland

**Keywords:** *Dermestes*, forensic entomology, indoor, larder beetles, mummification

## Abstract

**Simple Summary:**

Dermestidae are beetles of economic importance but also of significance for forensic entomology. In the latter case, Dermestidae are classified as colonisers of cadavers in late stages of decomposition when the remains are in a dry phase (mummified human corpses) or are partially skeletonised. Therefore, they are classified as secondary necrophages. This article describes three cases of mummified human corpses in indoor conditions that were colonised by representatives of the Dermestidae. All three cases are linked by the presence of *Dermestes* (*s.str.*) *haemorrhoidalis*—a species considered relatively rare in Poland, whose presence on human corpses was first observed in 2020 in Wroclaw.

**Abstract:**

Beetles of the family Dermestidae, especially of the genus *Dermestes* Linnaeus, 1758, are often identified on corpses in late stages of decomposition. They usually feed on remains devoid of organs and soft tissues or when the corpses undergo mummification. In Europe, eight species from two subgenera *Dermestes* and *Dermestinus* Zhantiev, 1967, have so far been identified on human corpses. Despite the relatively frequent presence of *Dermestes* sp. in experimental studies conducted in Poland, no reports concerning *Dermestes* directly collected from human corpses have been published to date. This article again describes observations of Dermestidae collected from human corpses found in indoor conditions in Wrocław, the capital of the Dolnośląskie Voivodeship. For the second time, there is evidence of the presence of *Dermestes* (*s.str.*) *haemorrhoidalis* on human corpses—a species considered to be relatively rare, as evidenced by faunistic data published from Poland, as well as the results of ongoing experiments of forensic interest.

## 1. Introduction

Beetles of the genus *Dermestes* Linnaeus, 1758 (Coleoptera: Dermestidae) are necrophagous insects found on corpses, including human corpses [1,2,3,4]. Their most intense activity occurs during late stages of decomposition—for this reason, they are classified as secondary necrophages. These insects prefer dry and dehydrated remains. The consumption The consumption of dried substrate reduces competition on corpses by limiting this food resource to adapted species. The morphology and biology of selected species were presented by Mroczkowski [5] and Peacock [6]. Information on larval stages was collected by Peacock [6] and Kadej [7]. Currently, the genus *Dermestes* includes approximately 90 species, including fossils ones [8]. Out of this number, as a direct result of their observation on human corpses, only a few taxa are classified in Europe as species of forensic interest [1,3,4,9]. Reviews were recently published on the subject [1,10]. An example of such species is *Dermestes* (*s.str.*) *haemorrhoidalis* Küster, 1852. This species, described from southern France, mainly inhabits southern Europe while data on its distribution are fragmentary, especially in central Europe. Moreover, this species has been reported in Africa (Burundi, Congo, Madagascar, South Africa, Tanzania, Zambia), South America (Argentina, Bolivia, Brazil, Chile, Peru, Uruguay), and Asia (China, Iran, Japan, Mongolia, Oman, Russia, South Korea, Vietnam) [8]. In countries with hot climates, this species is classified as a pest in warehouses and stores of fur and leather products, cheese, and dried meat. This species produces two generations per year. Both larvae and adults feed on dried meat, leather, cheese, dead insects, and other dry animal substances. In order to build a pupal chamber, adult larvae munch their way through harder materials such as wood, cork, and pressed tobacco, thus causing additional damage to the storage rooms [5,6,11,12]. Information regarding the biology and economic importance is provided by studies by Mroczkowski [5] and Peacock [6]. Studies on the length of the development cycle under varying conditions of temperature and relative humidity were in turn published by Coombs [13] and Jakob and Fleming [14]. In Poland, *D.* (*s.str.*) *haemorrhoidalis* was first found in Warsaw, where it was brought in the 1960s and where it probably acclimatised [5], as evidenced by individuals collected in the Warsaw Śródmieście District in 1978 and 1981 [15]. The distribution of this species in Poland should be considered fragmentary. To date, this species has been reported in only a few locations, as evidenced by the small number of published faunistic data [16] and data from forensic publications [4].

This article describes below three cases of mummified human corpses from south-western Poland from the city of Wrocław, in which the presence of *Dermestes* (*s.str.*) *haemorrhoidalis* was reported.

In this article, the species of associated insects are only briefly described. This article includes comparisons of published data on *Dermestes* sp. found in Poland on animal or human corpses both under experimental conditions and in available case studies.

## 2. Materials and Methods

### 2.1. Materials

Insects were stored in plastic vials for morphological examination. The studied material was collected directly from the corpse in an autopsy room (cases #1–3) or at the place where the corpse was found (e.g., flat, case #1). Species identification was conducted using a Nikon SMZ800 (Tokyo, Japan) binocular microscope. All materials are stored at the Centre for Forensic Biology and Entomology, University of Wrocław.

### 2.2. Preparation

Both dead and live larvae/adults were preserved in 75% ethanol. Larval moults, after drying, were stored in plastic boxes. For final species identification, male genitalia were separated and dissected and then preserved in warm 10% KOH and washed in distilled water.

### 2.3. Identification

Species identification was conducted using a Nikon SMZ800 (Tokyo, Japan) binocular microscope. The identification of Dermestidae was made by the analysis of specific species characteristics of both adults and larvae, including the comparison of male genitalia [5,6,17]. Other references were used for the identification of Calliphoridae [18] and Piophilidae [19,20].

### 2.4. Light Photography and Image Processing

Images were taken under a Nikon SMZ800 (Tokyo, Japan) binocular microscope with a Nikon Coolpix^®^ 4500 (Tokyo, Japan) digital camera. Image stacks were processed using Combine ZP^®^ software (Derby, United Kingdom).

## 3. Results

### 3.1. Case 1

**Figure 1:**
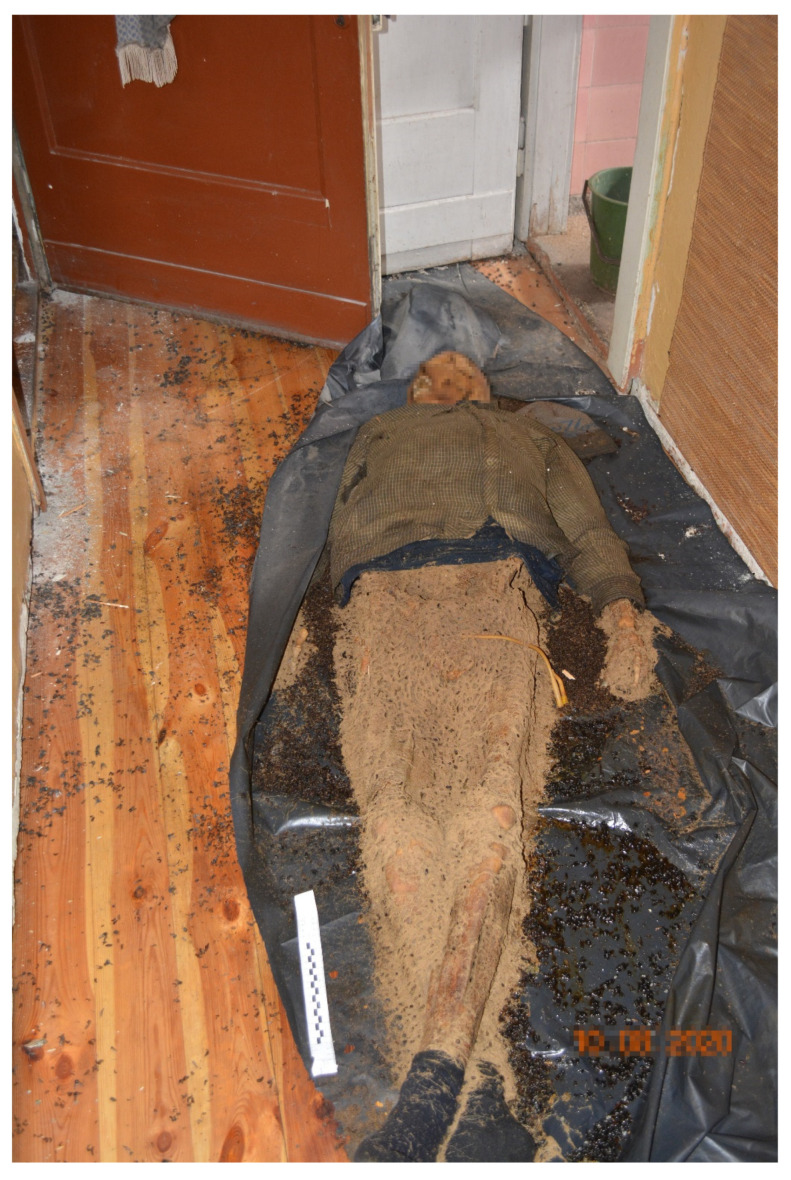
View of the corpse at the scene of its discovery (case #1).

**Figure 2:**
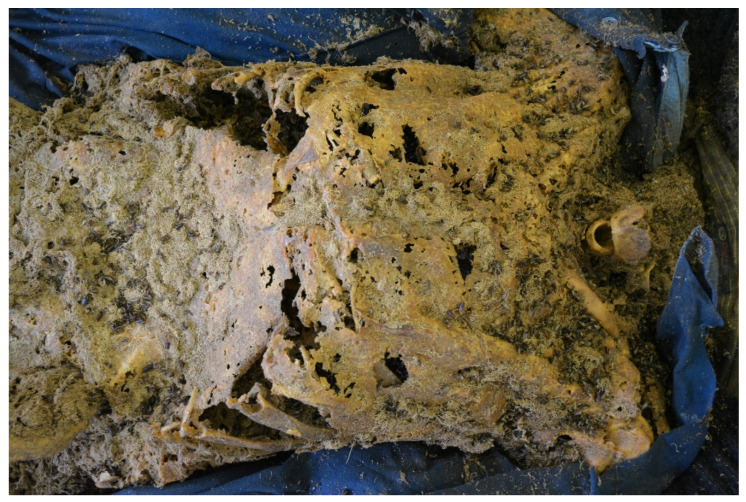
Feeding marks of *Dermestes* sp. (Coleoptera: Dermestidae) on the trunk of the cadaver (case #1).

On 10 August 2020, the corpse of an 83-year-old single man was discovered in a flat on the first floor of a multifamily two-story house in an old (before World War II) building in the centre of a large city (Figure 1). The corpse lay on the floor in the hallway near the entrance. The deceased lay on his back on an unfolded plastic sheet. He suffered from prostate cancer. The man was last seen by his neighbours around Christmas 2019. The flat was found to contain shopping receipts, postal orders, and a Housing Association document dated February 2020. The forensic post-mortem examination revealed advanced late post-mortem changes in the form of skeletonisation of the corpse with residually preserved and transformed–mummified-soft tissues (integuments and ligaments) and fragments of internal organs (dura mater, brain tissue, and pericardial sac). The weight of the corpse was approximately 24 kg. The skull and three upper cervical vertebrae were separated from the body (Figure 2). The integuments were yellowish-brown, dry, constricted, and brittle with numerous patchy defects and holes with uneven jagged edges (feeding marks of beetles). Beetles of the family Dermestidae were present on the corpse at various developmental stages, as well as frass (loose, weighing approximately 372 g) and puparia of Diptera. Other findings included a Foley catheter in the cadaver’s pelvis; degenerative changes of the left knee joint; status post a healed skull bone fracture; sequelae of neurosurgery and of orthopaedic surgery of the left lower leg; and dental cavities and fillings. The cause of death was not determined.

**Figure 3:**
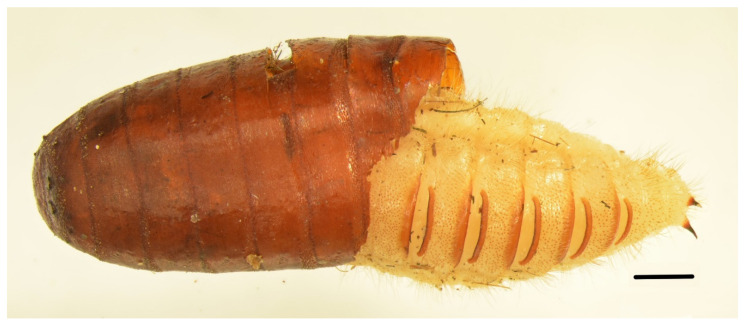
A pupa of *D.* (*D*.) *frischii* in a puparium, which was collected from the site where the corpse was found and preserved, i.e., in the flat of the deceased (case #1, scale = 1 mm).

In an autopsy room, mostly dead adults, larvae, and larval moults of two Dermestidae species were collected from the corpse and preserved, i.e., *D.* (*Dermestinus*) *frischii* Kugelann, 1792 (Coleoptera: Dermestidae) (Figure 3) and *D.* (*s.str.*) *haemorrhoidalis* (Coleoptera: Dermestidae). Adults of three Dermestidae species of the genus *Dermestes* (Coleoptera: Dermestidae) were collected from the flat of the deceased and preserved. They included *D.* (*D.*) *frischii* (hundreds of individuals), *D.* (*s.str.*) *lardarius* Linnaeus, 1758 (Coleoptera: Dermestidae) (six individuals), and *D.* (*s.str.*) *haemorrhoidalis* (17 individuals). *Attagenus smirnovi* Zhantiev, 1973 (Coleoptera: Dermestidae) (multiple live larvae) and *Anthrenus verbasci* (Linnaeus, 1767) (Coleoptera: Dermestidae) (one live larva) were also collected from the flat and preserved. Puparia and dead adults of flies *Calliphora vicina* Robineau-Desvoidy, 1830 (Diptera: Calliphoridae), *Lucilia sericata* (Meigen, 1826) (Diptera: Calliphoridae), and dead adults of *Sarcophaga* Meigen, 1826 (Diptera: Sarcophagidae) were present in large numbers on windowsills, furniture, and the floor (16 individuals).

### 3.2. Case 2

**Figure 4:**
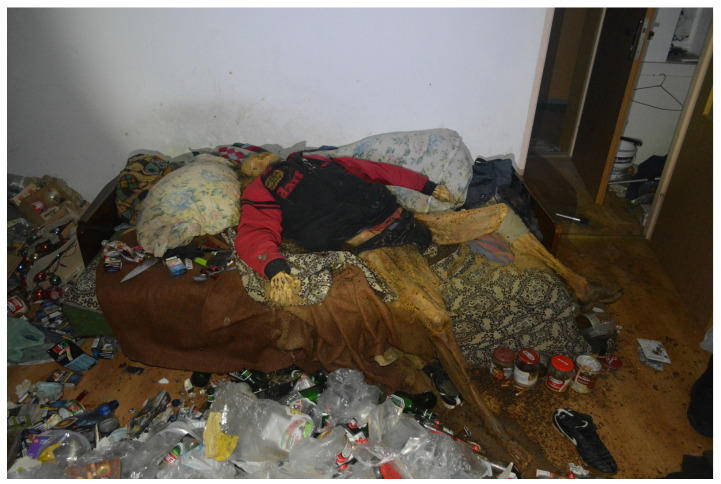
View of the corpse at the scene of its discovery (case #2).

**Figure 5:**
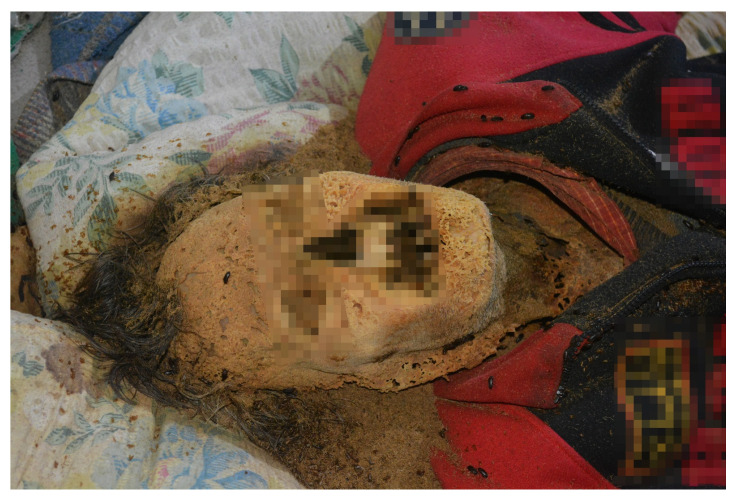
Feeding marks of *Dermestes* (*s.str.*) *haemorrhoidalis* on the head of the corpse (case #2).

On 10 May 2021, the corpse of a 60-year-old single man was discovered in a flat in a large-panel 1960s multistorey block of flats in the centre of a large city. The deceased lay on his back on a fold-out sofa (Figure 4). There was a mess in the room where the body was found. In the immediate vicinity of the corpse, there were numerous beer cans, bottles, plastic bags, empty plastic food packaging, empty metal cans, cigarette butts, etc. Neighbours and acquaintances had last contact with the deceased about six months earlier. The forensic post-mortem examination revealed advanced late post-mortem changes in the form of natural mummification and partial skeletonisation of the corpse. There was a residual preservation of the following internal organs: a transformed brain with completely blurred structure and a conglomerate of transformed internal organs of the thorax and abdominal cavity, which were impossible to identify. The weight of the corpse was approximately 24 kg. Head and facial integuments were dried with bones (Figure 5). The soft tissue was hard, dry, brownish-yellow, dull, rough, and greasy on the outer surface with numerous holes and cavities—0.3 to 2.0 cm in diameter—with even, slightly rounded edges. At the bottom of the cavities, there were exposed skull bones and entomological material. Skin on the trunk and limbs was yellowish-brown and light brown with a patchy pattern, hardened, dry, dull, rough, greasy on external surface, dried with bones, and wrinkled; it was also covered with entomological material and cavities of various sizes with uneven, jagged, brittle and yellow-coloured edges filled with entomological material (evidence of beetle feeding). Moreover, the following findings were made: degenerative changes in the joints of the left wrist; degenerative and proliferative changes of the lumbar spine in the form of osteophytes; a healed fracture with displacement of the proximal epiphysis of the right humerus; sequelae of traumatic injury of the right shoulder joint with deformation of the clavicle and scapula; a healed fracture of the proximal epiphysis of the left tibia with the presence of an orthopaedic plate; and a healed fracture of the fifth bone of the right metacarpus. The cause of death was not determined.

In terms of insects found on the corpse, adults and larvae of *D.* (*s.str.*) *haemorrhoidalis* (hundreds of individuals) were collected and preserved. In this case, the forensic entomologist was not present at the site where the body was found.

### 3.3. Case 3

**Figure 6:**
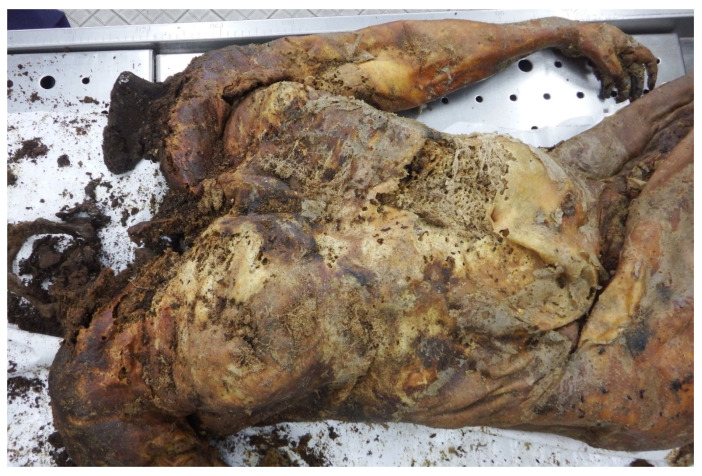
State of preservation of the corpse with obvious damage by *Dermestes* (*s.str.*) *haemorrhoidalis* (case #3).

On 26 February 2021, the corpse of a 75-year-old single woman was discovered in a flat on the fifth floor of a block of flats dated around 2000. The deceased lay on her back on the kitchen floor; the flat was in disarray. The woman did not maintain contact with her family and neighbours. In March 2019, there was an emergency room intervention for her due to cardiac problems. A neighbour living opposite to the flat where the body was found last saw the deceased about two years earlier; he used to see her very rarely. Autopsy findings included advanced late post-mortem changes in the form of natural mummification with soft tissue loss involving the head, neck, shoulders, and upper thoracic region. The residual preservation of internal organs included a transformed brain with completely blurred structure and a homogeneous conglomerate of highly transformed trunk organs that were impossible to identify. Insects and evidence of their development and feeding (Coleoptera and Diptera) were found on the corpse (Figure 6). The weight of the corpse was approximately 30 kg. The skull was completely separated from the body. Integuments were yellowish brown to dark brown, dry, hardened, and greasy on the outer surface with numerous cavities with even and uneven jagged and brittle edges. The cause of death was not determined.

*Necrobia rufipes* (De Geer, 1775) (Coleoptera: Cleridae) (eighty-five adults and twenty larvae) and *D.* (*s.str.*) *haemorrhoidalis* (twenty-two adults, many larval moults, and five larvae) were collected from the corpse in an autopsy room and preserved. In addition to beetles, developmental forms of the following Diptera were collected: *Piophila casei* (Fallén, 1813) (Diptera: Piophilidae) (one larva, twenty-six puparia, and two adults), puparia of Phoridae (probably *Megaselia* sp. Rondani, 1856, eleven puparia), and *Chrysomya albiceps* (Wiedemann, 1819) (Diptera: Calliphoridae) (numerous puparia and three dead adults). In this case, the forensic entomologist was not present at the site where the body was found.

## 4. Discussion

In Europe, eight species of the genus *Dermestes* were identified on human corpses: *D*. (*Dermestinus*) *frischii*; *D*. (*D*.) *maculatus* DeGeer, 1774; *D*. (*D.*) *undulatus* Brahm, 1790; *D*. (*s.str*.) *ater* DeGeer, 1774; *D*. (*s.str*.) *bicolor bicolor* Fabricius, 1781; *D*. (*s.str*.) *haemorrhoidalis*; *D*. (*s.str*.) *lardarius;* and *D*. (*s.str*.) *peruvianus* Laporte de Castelnau, 1840 [1,4,6]. The above-mentioned *D*. (*s.str*.) *haemorrhoidalis* was mainly found on dry or skeletonised corpses decomposing in indoor conditions [1]. With regard to data from Poland, the first published identification of this species on a human corpse came from Wrocław, also from the mummified corpse of a man found in a flat in one of the city’s tenement buildings [4]. In this particular case, *D*. (*s.str.*) *haemorrhoidalis* was the only representative of the genus *Dermestes,* represented by larval moults, live larvae, dead and live adults, and frass. Similarly, in the three cases presented in this paper, *D*. (*s.str*.) *haemorrhoidalis* was the species found on the corpses and was even the dominant species (cases #2, 3). All of the above-mentioned cases of corpse mummification took place in the urban agglomeration and inside living quarters.

Case #1, which was described in this paper, had a composition of insect species found on corpses that was strongly similar to the assemblage reported from Wrocław by Kadej et al. [4]. The difference is that in addition to species such as *A. smirnovi*, *A. verbasci,* and *C. vicina*, the following species were also collected from the flat and corpse and preserved: *D.* (*D.*) *frischii*, *D.* (*s.str.*) *lardarius*, *L. sericata,* and *Sarcophaga* sp. As the study by Lutz et al. proved [21], Diptera such as *L. sericata* and *C. vicina* and beetles such as *D.* (*s.str.*) *lardarius* were the most abundant species on human corpses analysed in Germany. In case #2, the species composition was dominated by *D*. (*s.str*.) *haemorrhoidalis*. This situation can be explained by specific circumstances, e.g., the hot and dry environment, rapid mummification of the body, and the absence of other necrophagous insects. In such conditions, *Dermestes* sp. can colonize a body in huge numbers [21,22]. In case #3, the species composition was different and in addition to *D.* (*s.str.*) *haemorrhoidalis* still included species such as *N. rufipes*, *P. casei*, Phoridae (probably *Megaselia* sp.; Diptera), and *Ch. albiceps*. In the light of published data from Europe, the above-mentioned assemblage is fairly typical of indoor decomposition, excluding Piophilidae, which are considered rather typical of outdoor decomposition [21], as confirmed by field studies from Poland [23,24,25,26,27]. *Chrysomya albiceps*, although usually found in environments such as meadows and forests [3,24,28,29], was also found on corpses in indoor conditions [21,28,30]. Similar habitat preferences are shown by Phoridae [21,31,32]. *Necrobia rufipes* can also be found on both animal [23,26,27] and human corpses [21,33]. Observations of *N. rufipes* in relation to Dermestidae are also consistent with the results of a study by Charabidze et al. [1], which clearly revealed that one of the most common coleopteran species associated with dermestids were *Necrobia* spp., although it was observed especially for outdoor varieties.

In the described cases, only in case #1 there was more than one representative of *Dermestes* (three species) on the corpse. In the other two cases (cases #2–3), only *D.* (*s.str.*) *haemorrhoidalis* was found on the corpses. These observations are consistent with the results from northwestern Europe, where the highest percentage of findings was for only one species of *Dermestes* sp. on corpses, regardless of whether decomposition occurred in indoor or outdoor conditions [1].

Data from Poland (Table 1) from experiments on the decomposition of domestic pig (*Sus scrofa domestica* Linnaeus, 1758) carcasses in outdoor conditions detected the following representatives of the genus *Dermestes*: *D*. (*D*.) *frischii* [23,24,26,27,34], *D*. (*D*.) *murinus murinus* Linnaeus, 1758 [23,26,34,35,36], and *D*. (*D*.) *laniarius laniarius* Illiger, 1802 [26,34]. On the other hand, *D*. (*s.str*.) *lardarius*, *D*. (*D*.) *frischii*, and *D*. (*D*.) *undulatus* were also found on a male corpse in outdoor conditions [33] (Table 1). A specific case involved the identification of *D*. (*s.str*.) *lardarius* on a corpse pulled from a chimney [37]. The latter case is quite debatable regarding the problem of attribution to the conditions under which the decomposition occurred. However, given the height of chimney of 250 m, it can be assumed that these conditions were close to indoor ones. The above-mentioned comparison (Table 1) clearly shows that *D*. (*s.str*.) *haemorrhoidalis* prefers corpses in indoor conditions, such as rooms in residential buildings. This is also confirmed by previously published faunistic data from Poland [16] and the case from Wrocław that was described by Kadej et al. [4]. This situation can probably be explained by thermal requirements and the preference for a warmer climate [5]. Ruta et al. [16] found this species also in a large city, namely, Poznań—in each of the two cases, this species was found in enclosed spaces that were probably heated or thermally insulated during the winter. Interestingly, in the case from Poznań [16], this species was collected in a room together with *D.* (*s.str*.) *bicolor bicolor.* In one of the three cases described in this study (case #1), this species was in turn accompanied by *D.* (*D*.) *frischii* and *D.* (*s.str*.) *lardarius*. The latter observation partly coincides with the result obtained by Voigt [38], where *D.* (*s.str*.) *lardarius* was present on a corpse together with *D.* (*s.str*.) *haemorrhoidalis*.

In contrast to results from northwestern Europe where *D*. (*s.str*.) *peruvianus* was the dominant species on corpses in indoor conditions [1], observations of this study indicate that in the case of Wrocław, a city located in southwestern Poland, it is likely that the dominant species is in turn *D.* (*s.str.*) *haemorrhoidalis* [4]. Confirmation of this assumption, however, requires further research and analysis of cases from other locations in the Dolnośląskie Voivodeship and preferably elsewhere in Poland. Such material is currently being collected. If the relevant prosecutorial consents are obtained, the results will be published, representing—as it is assumed—a major addition to a better understanding of the Dermestidae and possible collected and preserved insect assemblages found on human corpses in Poland.

In all three cases described in this study (cases #1–3), there was only the damage to the dehydrated skin typical of Dermestidae (cf. [4]). Only in case #1 there was frass—fibrous horsehair-like, dark-brown material covering the body—a typical sign of feeding activity for larvae found on corpses [4,22,38]. Although the literature indicates the possibility of damage to the bones by larvae of the last stage, which penetrate the bones to make pupal chambers (which was experimentally confirmed with *D*. (*s.str*.) *maculatus* [9]), no such damage was found in any of the cases described in this study. Recent results show that the presence of pupal chambers in the bones depends on bone dryness and larval density [9]. The lack of bone damage in the cases described above, even if they were adequately dried, may be explained by the fact that in the case of corpses decomposing in a flat, larvae have the opportunity to penetrate spaces other than a corpse that are more suitable for the creation of pupal chambers. A flat offers a wide variety of opportunities in the form of numerous materials, such as paper, wood, plasterboard, and leather products—definitely softer materials than a human bone.

An interesting observation was made in case #1, namely, the confirmation of pupation, this time by *D.* (*D.*) *frischii*, in the empty shells of pupae of flies of the family Calliphoridae (Figure 3). A similar behaviour was described in the example of *D.* (*s.str*.) *haemorrhoidalis*, also collected from a mummified corpse found in one of the flats in Wrocław [4]. The evidence supports the thesis of the possible use of structures such as puparia as substitutes for pupal chambers that are created in harder material. Perhaps such behaviour is typical of numerous Diptera assemblages (high availability of empty puparia for Dermestidae larvae) and with higher densities of Dermestidae larvae (larval competition for space to pupate). It is also possible that *D*. (*D*.) *frischii* exhibits predatory behaviour in relation to larvae of Diptera, as experimentally proven for *D*. (*s.str*.) *ater* in relation to larvae of *Musca domestica* Linnaeus, 1758 (Diptera: Muscidae) [39]. These observations encourage further data collection in this area for other *Dermestes* sp. found on human corpses.

A separate commentary is required regarding the interpretation of the result of the presence of *D.* (*s.str*.) *haemorrhoidalis* in all so far published descriptions of mummified human corpses in indoor conditions in Wrocław ([4] and in this study). Flats provide the perfect place for this species to develop, enabling it to survive especially during the winter. It should also be mentioned that, according to Jacob and Fleming [14], the optimum temperature for development of this species is 20 °C, while eggs do not develop below 15 °C. In the case of living quarters, it is difficult to exceed the above-mentioned limit temperature even in winter, especially in view of recent winters in Wrocław that have been relatively warm and mild. Observations of *D.* (*s.str*.) *haemorrhoidalis* on corpses prove that this species, contrary to what Mroczkowski wrote in 1975 [5], maintains local populations in Poland. Although this species is not found en masse in flats compared to warehouses and storage rooms where it is a typical pest, it can be assumed that it invades premises when attracted by the smell of corpses from other spaces. Nesting sites of birds such as *Columba livia* f. *urbana* Gmelin, 1789 (Aves: Columbiformes: Columbidae), whose nests are very frequently built in residential buildings in Wrocław (attics, lofts, balconies, etc.), may be one of such sources. The possibility of the colonisation of bird nests by this species was mentioned by Hockin [40], as was also suggested as early as 1963 by Bezant [41], who wrote that there were indications that *D.* (*s.str*.) *haemorrhoidalis* might breed in pigeon nests in London. From bird nesting sites, *D. haemorrhoidalis* may be attracted to corpses not only by the smell of decaying remains, but also by a male-produced aggregation pheromone [42].

## 5. Conclusions

*Dermestes* (*s.str*.) *haemorrhoidalis* has so far been found sporadically in Poland, and knowledge of its distribution requires further in-depth faunistic studies. The results of autopsies of mummified human corpses, when confronted with data from field experiments, support the thesis that this is a species which mainly prefers decomposing corpses in indoor conditions. The cases presented by us indicate that *D*. (*s.str*.) *haemorrhoidalis* in this part of Poland may be a common or even the only larder beetle on corpses in the late stage of decomposition. This indicates the potential of this species in a forensic context. For this reason, further studies regarding the biology of this species found on human corpses would also be advisable, including studies in terms of its interactions with other species. According to recent scientific reports, it is also worth investigating the potential use of bones as material for the creation of pupal chambers by this species. The results described above also reveal the need to collect and preserve entomological material not only from human corpses in an autopsy room, but also directly at the site where the corpse was found. A visit by an entomologist to the site where a corpse was found not only allows more entomological evidence to be preserved, but also gives the opportunity to gather more information on species of forensic importance, their behaviour, and their use of the space around the corpse. For this reason, it should be good practice to allow a forensic entomologist or other competent person to participate in the examination of the body.

In the future, based on cooperation between institutions such as the police and the prosecutor’s office, the intention is to focus on Dermestidae that colonise human corpses in order to carefully analyse their communities in both outdoor and indoor conditions in Poland. This is particularly necessary because despite the relatively frequent occurrence of Dermestidae on human corpses, few studies analyse the communities of Dermestidae in detail.

## Figures and Tables

**Table 1:** The comparison of literature data regarding *Dermestes* Linnaeus, 1758 (Coleoptera: Dermestidae) on human corpses from Poland.

Taxa	Experiment/Case	Outdoor/Indoor	References
*D*. (*s.str*.) *haemorrhoidalis*	Case	Indoor (flat)	[4] and cases #1–3 in the current paper
*D*. (*s.str*.) *lardarius*	Case	Outdoor (forest habitat)	[33]
	Case	Indoor? (inside a chimney)	[37]
	Case	Indoor (flat)	Current paper (case #1)
*D*. (*D*.) *frischii*	Experiment with domestic pig carcasses	Outdoor (forest habitat)	[23,24]
	Experiment with domestic pig carcasses	Outdoor (open grassland habitat)	[26,27,34]
	Case	Outdoor (forest habitat)	[33]
	Case	Indoor (flat)	Current paper (case #1)
*D*. (*D*.) *laniarius laniarius*	Experiment with domestic pig carcasses	Outdoor (open grassland habitat)	[26,34]
*D*. (*D*.) *murinus murinus*	Experiment with domestic pig carcasses	Outdoor (forest habitat)	[23,24,35,36]
		Outdoor (open grassland habitat)	[34]
*D*. (*D*.) *undulatus*	Case	Outdoor (forest habitat)	[33]
*Dermestes* sp.	Experiment with domestic pig carcasses	Outdoor (forest habitat)	[25]

## Data Availability

The authors confirm that the data supporting the findings of this study are available within the article.
